# Aprepitant Inhibits JNK and p38/MAPK to Attenuate Inflammation and Suppresses Inflammatory Pain

**DOI:** 10.3389/fphar.2021.811584

**Published:** 2022-01-11

**Authors:** Yang Yang, Wei Zhou, Xiuqi Xu, Xianxiu Ge, Fei Wang, Guang-Qin Zhang, Lin Miao, Xueting Deng

**Affiliations:** ^1^ Medical Center for Digestive Diseases, Second Affiliated Hospital, Nanjing Medical University, Nanjing, China; ^2^ Department of Child Health Care, Children’s Hospital of Nanjing Medical University, Nanjing, China; ^3^ Department of Clinical Pharmacy, China Pharmaceutical University, Nanjing, China

**Keywords:** aprepitant, JNK, p38, NFκB, inflammatory pain

## Abstract

Substance P contributes to the pathogenesis of pain by acting on NK-1R, specialized sensory neurons that detect noxious stimuli. Aprepitant, an antagonist of NK-1R, is widely used to treat chemotherapy-induced nausea and vomiting. In this study, we used LPS-stimulated BV-2 microglia cell line and animal models of inflammatory pain to explore the analgesic effect of aprepitant on inflammatory pain and its underlying mechanism. The excitability of DRG neurons were measured using whole-cell patch-clamp recordings. The behavioral tests were measured and the morphological changes on inflamed paw sections were determined by HE staining. Changes in the expressions of cytokine were measured by using real-time quantitative PCR analysis and ELISA method. Immunofluorescence and western blotting were used to detect the microglia activation and MAPK. Aprepitant treatment significantly inhibited the excitability of DRG neurons. The pain behavior and the paw tissues inflammatory damage were significantly relived after the administration of aprepitant compared to formalin group. Aprepitant significantly suppressed the activation of microglia, phosphorylation of JNK and p38 MAPK, as well as the mRNA and protein expressions of MCP-1, TNF-α, IL-6, and IL-1β, *in vivo* and *in vitro*. The LPS-induced over-translocation into nucleus of NF-κBp65 was down-regulated following aprepitant treatment in BV-2 cells. The present study suggests that aprepitant attenuates inflammatory pain in mice via suppressing the phosphorylation of JNK and p38, and inhibiting the NF-κB signaling pathway.

## Introduction

Inflammatory pain is a common chronic pain in clinical, which adversely impacts the quality of life (Tomic et al., 2018; Gomes et al., 2020). Although to relieve inflammatory pain, significant progress has been achieved in existing analgesics, such as nonsteroidal anti-inflammatory drugs, opioid analgesics, the insufficient efficacy and adverse side effects limit the curative effect (Tomic et al., 2018; Gomes et al., 2020). Consequently, promising analgesic agents against inflammatory pain are imminently required.

Mechanisms of inflammatory pain are complex and involve many factors. It has long been generally accepted that microglia in spinal cord play an important role in the pathogenesis of inflammatory pain. Spinal cord microglia are persistently activated in inflammatory pain conditions (Wolf et al., 2017; Chen et al., 2018). Upon activation, microglia secrete numerous inflammatory mediators, such as tumor necrosis factor-α (TNF-α), interleukin-1β (IL-1β), interleukin-6 (IL-6), and monocyte chemoattractant protein 1 (MCP-1) (Wolf et al., 2017; Zhou et al., 2018). These proinflammatory mediators can damage surrounding neurons and enhance the hyperactivity of neurons, resulting in central sensitization and enhanced pain sensitivity (Ji et al., 2018). Therefore, inhibiting the inflammatory microglial response will be beneficial for inflammatory pain therapy. Numerous studies have recently indicated that minocycline (a microglial inhibitor) significantly suppresses the behavioral and neurochemical signs of chronic pain (Cho et al., 2006; Mika et al., 2010; Zhou et al., 2018). However, minocycline, as a broad-spectrum antibiotic, its prolonged use may cause severe side effects including systemic lupus erythematosus-like syndrome and autoimmune hepatitis (Gough et al., 1996). Therefore, a novel, potent and safe drug is urgently needed for the treatment of inflammatory pain through microglial inhibition.

Aprepitant, a highly selective and non-peptide neurokinin-1 receptor (NK-1R) antagonist, is approved for the prevention of chemotherapy-induced nausea and vomiting by the U.S. Food and Drug Administration. Aprepitant is safe and well tolerated for currently used in clinical practice. Furthermore, it has been reported that aprepitant plays specific anti-inflammatory roles in HIV infection (Wang et al., 2008; Tebas et al., 2015), osteoarthritis pain (Ogawa et al., 2016), rheumatoid arthritis (Liu X. et al., 2019), prurigo nodularis (Agelopoulos et al., 2019). This drug exerts anti-inflammatory, analgesic, antiviral, and antiemetic effects (Liu BK. et al., 2019; Munoz and Covenas, 2020).

A previous study has reported that aprepitant can act as an anti-inflammatory agent and attenuate lipopolysaccharide (LPS)-induced inflammatory responses in macrophages (Zhao et al., 2020). Aprepitant not only significantly suppresses HIV-1 infection of microglia (Wang et al., 2008), but also downregulates microglia M1 markers, and upregulates microglia M2 markers following intracerebral hemorrhage (Jin et al., 2021). However, there is currently no research on the role of aprepitant in inflammatory pain. Therefore, we hypothesized that aprepitant might provide benefit for treatment of inflammatory pain, and interfere with microglia activation, as well as the production of inflammatory cytokines in microglia. Hence, this research provides a novel view of the role of aprepitant in anti-inflammatory responses, which in turn provides a potential therapeutic approach for inflammatory pain.

## Materials and Methods

### Animals

The study (protocol code IACUC-2012045) was approved by the Ethics Committee of Nanjing Medical University. All experimental procedures were performed in accordance with the guidelines for laboratory animal care. Adult male ICR mice (6–8 weeks old, weight 18–22 g) were obtained from the Qinglongshan Animal Center of Nanjing (Nanjing, China), and kept in a temperature-controlled environment on a 12 h natural light-dark cycle with free access to food and water.

### Cells and Viability Assay

Microglia BV-2 cells were maintained in DMEM (Hyclone/Thermo), supplemented with 10% (v/v) FBS (Gibco), penicillin (100 U/ml) and streptomycin (100 μg/ml) (Hyclone/Thermo) in 5% CO_2_ at 37°C. Cells in logarithmic growth were used for experiments. Aprepitant (#HY-10052) was obtained from MedChemExpress. To assess cell viability, the BV-2 cells (5 × 10^4^ cells/mL) were seeded in 96-well plates. After treatment with aprepitant (1, 2, 4, 8 μM) for 24 h, 10 μL of MTT solution (5 mg/ml, Sigma-Aldrich, #M2003) was added to each well and the cells were incubated for 4 h. The supernatant was removed and 150 μL of DMSO (Sigma-Aldrich, #D4540) was added to dissolve formazan crystals in each well. After 30 min of incubation, absorbance levels for formazan at 570 nm were measured using a microplate reader.

### Mice Lumbar Dorsal Root Ganglion Cell Culture Preparation

Dorsal root ganglion (DRG) neurons were quickly isolated from spine and placed into ice-cold DMEM (Hong et al., 2020), then digested in collagenase (1 mg/ml, type II; Worthington, #LS004176) and 0.25% trypsin (Gibco, #25200–056), followed by addition of 0.25% trypsin inhibitor. Then the DRG neurons were triturated with Pasteur pipettes and centrifuged. The cell pellet was resuspended in 1 ml of Neurobasal media. Isolated neurons were plated onto poly-d-lysine (100 g/ml) (BBI Life Sciences, #E607014-0002) and laminin (1 mg/ml)-coated 35-mm tissue culture dishes containing B27 supplement (Gibco, # 17504044), l-Glutamine (Gibco, #10270106), and 1% penicillin-streptomycin (Hyclone/Thermo, #SV30010). Cells were used for patch clamp recordings at 24 h post dissociation.

### Electrophysiology

The excitability of DRG neurons were measured using whole-cell patch-clamp recordings (Hong et al., 2020). Small DRG neurons (<25 μm) were chosen in our study. The neurons in the two groups were administered vehicle (0.5% DMSO in saline) or aprepitant (2 μM). Voltages were recorded by using a HEKA EPC 10 amplifier (HEKA Instruments). The data were obtained and analyzed by Pulse software (HEKA Instruments). Signals were filtered at 3 KHz and sampled at 10 KHz. Electrodes were drawn from borosilicate capillary glass using a P-97 puller. Electrodes had a resistance of 2–5 MΩ when filled with intracellular solution. The pipette solution contained (in mM) KCl 140, MgCl_2_ 1, EGTA 5, Na_2_ATP 2, and HEPES 5 (pH = 7.2, adjusted with KOH). The external solution contained (in mM): NaCl 137, MgCl_2_ 1.2, KCl 5.4, NaH_2_PO_4_ 1.2, CaCl_2_ 1, HEPES 10, and glucose 10 (pH = 7.4, adjusted with NaOH). The action potentials (APs) were recorded under the current-clamp mode. APs were evoked by 1 s depolarizing current pulses from −10 up to 100 pA at 5 pA steps. The minimum current that triggered the first AP was defined as rheobase. To observe the excitability, DRG neuron was stimulated by 2 × rheobase.

### Formalin Test

Twenty microliter of saline or 2% formalin (in saline) was subcutaneously injected into the hind paw. Mice were randomly divided into five experimental groups: control (Ctrl), formalin, formalin + 5 mg/kg aprepitant, formalin + 10 mg/kg aprepitant, formalin + 20 mg/kg aprepatant. Aprepitant was intraperitoneal injected 30 min before injection of 2% formalin. Mice were monitored for time spent licking paw, and number of lick bouts, for 60 min post-injection by researchers blinded to experimental condition (Hunskaar and Hole, 1987; Alhadeff et al., 2018). All sessions were video-recorded. The time spent paw licking and biting was calculated in 5-min and recorded for 60 min. Additionally, acute (0–5 min) and inflammatory (15–45 min) phase pain responses were quantified.

### Carrageenan-Induced Inflammatory Pain

To establish the inflammatory pain model, 1% carrageenan (25 μL) was injected into the hind paw. Mice were randomly allocated to following groups: control, carrageenan, carrageenan + 5 mg/kg aprepitant, carrageenan + 10 mg/kg aprepitant, carrageenan + 20 mg/kg aprepitant. Aprepitant was intraperitoneal injected 30 min before injection of carrageenan.

### Immunohistochemistry and Inflammation Scoring

Paw tissues were quickly excised from deeply anesthetized mice, and then fixed in 10% formalin at 4°C. Briefly, sections were cut using a microtome, stained with HE, and visualized by the light microscope. The degree of inflammation was quantified using a 0 to five scoring system. The scores were defined as follows: 0 = no inflammation, 1 = mild inflammation, 2 = mild/moderate inflammation, 3 = moderate inflammation, 4 = moderate/severe inflammation and 5 = severe inflammation (Bang et al., 2009).

### Thermal Hyperalgesia

The instrument temperature was set at 55.0 ± 0.5°C (Alhadeff et al., 2018; Qin et al., 2019). Mice were placed separately on the heated surface. The reaction time for mice contraction or licking hind paws is paw withdrawal latency. A cut-off time of 25 s was set to avoid tissue damage. All testing was conducted blindly with respect to group assignment.

### Mechanical Allodynia

Mechanical allodynia was indicated by von Frey filaments ranging from 0.02 to 6 g (Alhadeff et al., 2018). After 30 min of adaptation, filaments were used to stimulate the plantar surface of each hind paw. The filaments were applied to six designated loci distributed over the plantar surface of the hind paw. Withdrawal threshold was determined as the filament at which the mouse responded with a withdrawal response, including licking, paw withdrawal, and trembling, to > 50% of trials.

### ELISA

The spinal cord at L4-L5 segments was quickly removed from deeply anesthetized mice. Whole-cell lysates were harvested using RIPA buffer and centrifuged for 15 min at 12,000 rpm at 4°C, and then the supernatant was transferred to a new tube. The levels of TNF-α, IL-1β, IL-6, and MCP-1 in the samples were determined using ELISA kits (Neobiocisence), according to the manufacturer’s instructions.

BV-2 cells were co-cultured with LPS (1 μg/ml) and aprepitant (2 μM) for 16 h. The levels of TNF-α, IL-1β, IL-6 and MCP-1 in the culture supernatant were analyzed by ELISA kits (Neobiocisence) following the manufacturer’s instructions.

### RNA Extraction and real-time PCR

Under deep anesthesia, the L4-L5 spinal cord segments s and the paw tissue samples of mice were quickly removed and analyzed. The dose of aprepitant used for the BV-2 cell culture was 2 μM. Total RNA was extracted from tissues or cells with TRIzol reagent and reversely transcribed using a reverse transcription kit (Thermo Fisher Scientific, #N8080234) according to the manufacturer’s instructions. The subsequent real-time PCR was performed with the SYBR^®^ Green qPCR Kit (Vazyme Biotech Co., #Q-311). The relative amount of gene expression was normalized to the endogenous control GAPDH. Differential expression was calculated according to the 2^−ΔΔCT^ method and statistically evaluated. The sequences of primers are listed in Table 1.

**TABLE 1:** The primer sequences used for real-time PCR.

Target Gene	Upstream Sequence (5ʹ-3ʹ)	Downstream Sequence (5ʹ-3ʹ)
MCP-1	5′-CTT CTG GGC CTG CTG TTC A-3′	5′-CAG CCT ACT CAT TGG GAT CA-3′
TNF-α	5′-CAT CTT CTC AAA ATT CGA GTG ACA A-3′	5′-TGG GAG TAG ACA AGG TAC AAC CC-3′
IL-6	5′-CCC TAC TTC ACA AGT CCG GAG AGG AGA-3′	5′-GGT AGC ATC CAT CAT TTC TTT GTA TCT CT-3′
IL-1β	5′-CCT GTG TCT TTC CCG TGG ACC TTC CAG G-3′	5′-CAT CAT CCC ATG AGT CAC AGA GGA TGG G-3′
GAPDH	5′-ACC ACA GTC CAT GCC ATC AC-3′	5′-TCC ACC ACC CTG TTG CTG TA-3′

### Western Blotting

Total proteins were isolated from the spinal cord at L4-L5 segments or the cell lines. The dose of aprepitant used for the BV-2 cell culture was 2 μM. Protein lysates were separated by SDS PAGE gels and then transferred to PVDF membrane (IPVH00010, Millipore). The membrane was incubated in 5% nonfat milk or 3% bovine serum albumin and then blotted with specific primary antibody. After incubation in the horseradish peroxidase (HRP)-conjugated secondary antibody, the membrane was detected with chemiluminescence western blot detection system (Bio-Rad Laboratories, CA, United States). The density was measured with ImageJ software (Fuji Film, Tokyo, Japan). Western blot antibody included rabbit-anti IBA1 (#016–20001, 1:1000 dilution, Wako), and rabbit-anti p-ERK (#4377, 1:1000 dilution), rabbit-anti ERK (#4695, 1:1000 dilution), mouse-anti p-JNK (#9255, 1:2000 dilution), rabbit-anti JNK (#9252, 1:1000 dilution), rabbit-anti p-p38 (#4511, 1:1000 dilution), rabbit-anti p38 (#9212, 1:1000 dilution), rabbit-anti p-NFκBp65 (#3033, 1:1000 dilution), mouse-anti NFκBp65 (#6956, 1:1000 dilution) which purchased from Cell Signaling Technology, and rabbit-anti GAPDH (#g9545, 1:6000 dilution, Sigma-Aldrich). HRP-conjugated anti-mouse (#12–349, 1:2000 dilution) and anti-rabbit secondary antibody (#12–348, 1:3000 dilution) were purchased from Sigma-Aldrich.

### Immunofluorescence

After washing with PBS, these slides were subsequently blocked with 3% BSA. The primary antibody, rabbit-anti IBA1 (#ab178847, 1:100 dilution, Abcam) or rabbit-anti NFκBp65 (#8242, 1:400 dilution, Cell Signaling Technology), was incubated overnight at 4°C, and then incubated with CyTM3-conjugated affiniPure Donkey Anti-Rabbit IgG (#711-165-152, 1:250 dilution) at room temperature for 2 h. The cells were then stained with DAPI. Images were acquired by confocal laser-scanning microscope (Leica, Lsm710).

### Statistical Analysis

The results were analyzed by GraphPad Prism and expressed as means ± SEM. Alteration of expression of the proteins or mRNA detected and the behavioral responses to mechanical stimuli over time among groups were tested with 1-way and 2-way ANOVA followed by Bonferroni post hoc tests, respectively. Statistical results are considered significant if *p* < 0.05.

## Results

### Aprepitant Inhibited the Excitability of Dorsal Root Ganglion Neurons and Suppressed Formalin-Induced Pain Behavior

**FIGURE 1:**
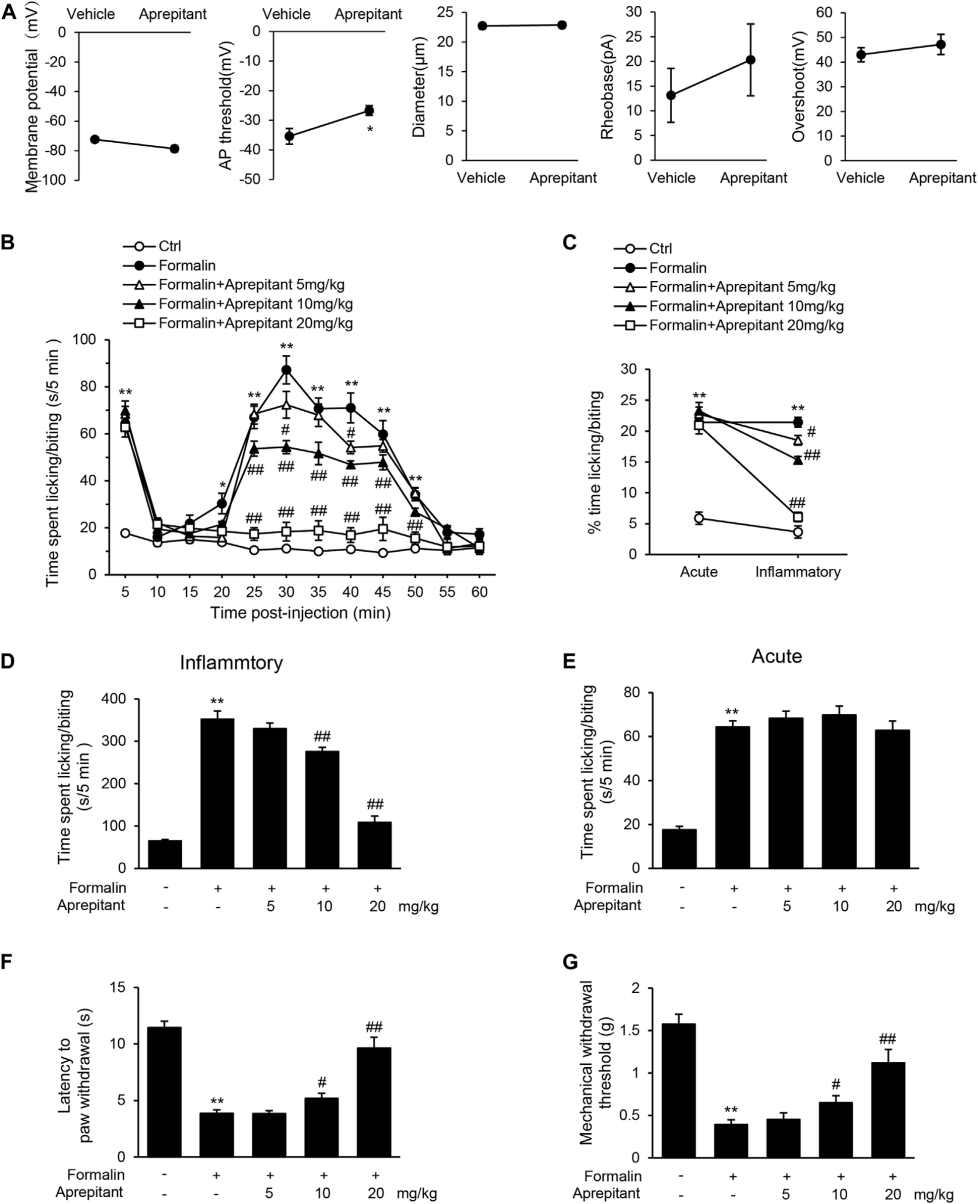
Aprepitant attenuated response to inflammatory pain. **(A)** Membrane properties of DRG neurons in control and aprepitant (2 μM) treatment groups. AP, action potential; Rheobase, the minimum current required to trigger an AP. Statistical significance was evaluate by the unpaired Student’s *t* test. **(B)** Time spent licking paw following formalin injection displayed in 5-min time bins after single injection of aprepitant. **(C)** % time spent paw licking during acute and inflammatory phases of formalin test with aprepitant treatment. **(D)** Time spent paw licking during the inflammatory phase of formalin test. **(E)** Time spent paw licking during the acute phase of formalin test. **(F)** Latency to withdraw paw in aprepitant treatment groups versus control mice during hotplate test. **(G)** Withdrawal threshold (Von Frey filament at which mouse responded to >50% of trials) in aprepitant treatment groups versus control mice. Twelve mice were included in each of the groups (*n* = 12). Data are expressed as mean ± SEM. Two-way ANOVA, *p < 0.05, **p < 0.01 versus vehicle or control, #*p* < 0.05, ##*p* < 0.01 versus formalin.

Substance P has been shown to participate in the inflammatory process (Suvas, 2017), and NK-1R is expressed in DRG neurons (Tang et al., 2008). To investigate the effect of aprepitant on the excitability of DRG neurons, we observed the characteristics of APs in dissociated nociceptive neurons (about 25 μm diameter). As shown in Figure 1A, aprepitant treatment showed a significantly higher AP threshold compared with control group *in vitro*. There were no significant differences in resting membrane potential, rheobase, and overshoot. These results demonstrated that aprepitant could decrease the excitability of DRG neurons. Intra-plantar injection of formalin induces distinct acute (0–5 min) and long-term inflammatory (15–45 min) phases in nocifensive behavior, while responses to hotplate or von frey filaments are acute and transient. We found that aprepitant treatment attenuated the pain behavior (Figures 1B–D, *p* < 0.01) of inflammatory paw licking/biting after injection of aprepitant. Conversely, aprepitant had no effect on the acute phase response to formalin injection (Figure 1E, *p* > 0.05). In addition, aprepitant alleviated the response to thermal (Figure 1F, *p* < 0.05) or mechanical (Figure 1G, *p* < 0.01) pain.

### Aprepitant Relieved Inflammation in the Mice With Formalin-Induced Inflammatory Pain

**FIGURE 2:**
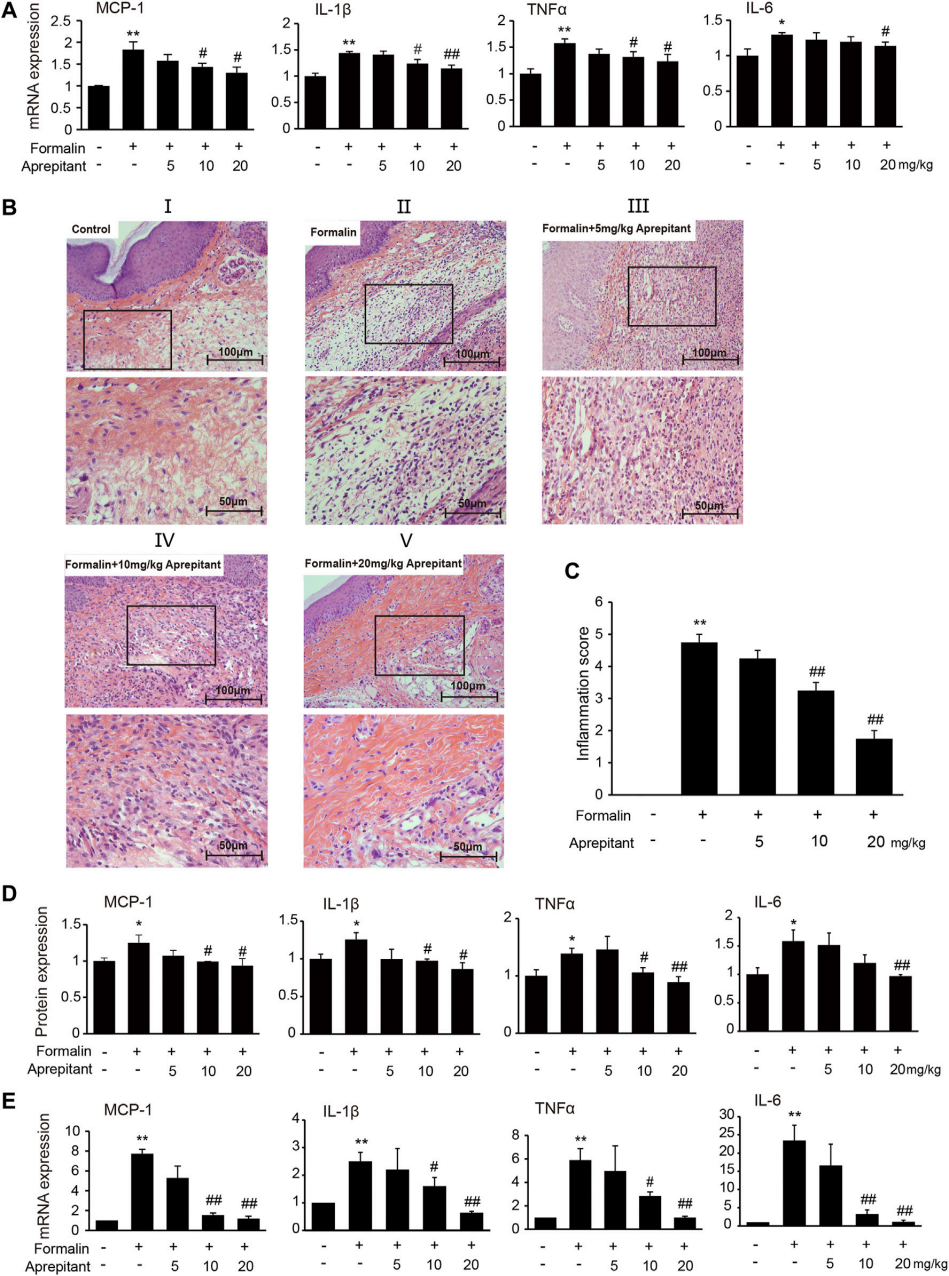
Anti-inflammatory effects of aprepitant in mice with formalin-induced inflammatory pain. **(A)** mRNA expression of MCP-1, IL-1β, TNF-α and IL-6 in the inflamed paw after administration of aprepitant. **(B, C)** Hematoxylin eosin (HE) staining (B) and the scores (C) of paw tissues of mice. Each photo is representative of six specimens for each group. **(D, E)** The protein (D) and mRNA (E) levels of MCP-1, IL-1β, TNF-α and IL-6 in the spinal cord of formalin treated mice after administration of aprepitant. Six samples were included in each of the groups (*n* = 6). Data are expressed as mean ± SEM. One-way ANOVA, *p < 0.05, **p < 0.01 versus control, #*p* < 0.05, ##*p* < 0.01 versus formalin.

To investigate the effect of aprepitant on proinflammatory cytokines, we measured the levels of MCP-1, IL-1β, TNF-α and IL-6 following formalin injection in mice. Administration of aprepitant (10 mg/kg, and 20 mg/kg) significantly inhibited the formalin-induced expression of MCP-1, IL-1β, TNF-α and IL-6 in the inflamed paw (Figure 2A, *p* < 0.05). Histopathological examination showed that the control group fed saline had normal paw tissue (Figure 2BI). In contrast, compared to the control group, the left hind paws of mice that received formalin injections showed massive accumulation of infiltrated cells (Figure 2BII). However, treatment with aprepitant (10 mg/kg, and 20 mg/kg) remarkably decreased inflammatory cell infiltration (Figure 2BIII–V). The inflammation scores indicated that co-treatment with aprepitant (10 mg/kg, and 20 mg/kg) significantly reduced formalin-induced inflammation (Figure 2C, *p* < 0.05). To further explore the anti-inflammatory effects of aprepitant, the levels of MCP-1, IL-1β, TNF-α and IL-6 in spinal cord were evaluated using ELISA and qRT-PCR. Compared with the control group, the protein and mRNA expressions of MCP-1, IL-1β, TNF-α and IL-6 were significantly increased in the formalin group (Figures 2D,E, *p* < 0.05). However, aprepitant remarkably reduced the expressions of these cytokines (Figures 2D,E, *p* < 0.05).

### Aprepitant Suppressed LPS-Induced Activation in BV-2 Microglial Cells

**FIGURE 3:**
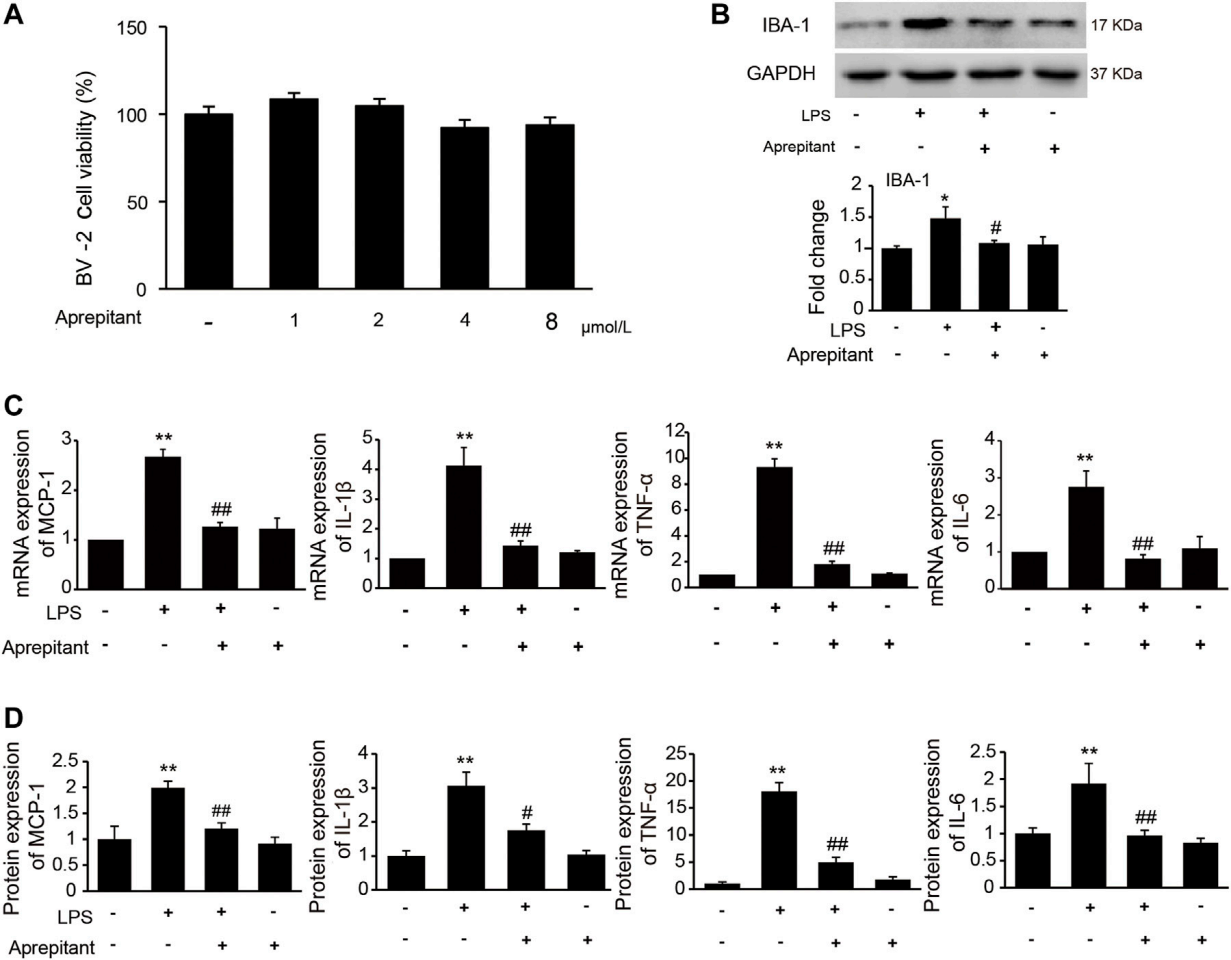
Effect of aprepitant on lipopolysaccharide (LPS)-induced BV-2 microglial activation. **(A)** Cell viability was measured using an MTT assay. **(B)** The representative immunoblots and graphic representation of relative expression of IBA-1. **(C)** The mRNA expression of MCP-1, IL-1β, TNF-α and IL-6 in BV-2 microglia. **(D)** ELISA showed that aprepitant decreased the LPS-induced secretion of MCP-1, IL-1β, TNF-α and IL-6 in BV-2 microglia (*n* = 6). Data are expressed as mean ± SEM. One-way ANOVA, *p < 0.05, **p < 0.01 versus control, #*p* < 0.05, ##*p* < 0.01 versus LPS.

It has been indicated that activation of microglia is crucial for the inflammatory stimuli, and involved in pain facilitation. To investigate the effects of aprepitant on LPS-induced microglia activation *in vitro*, we used the immortalized murine microglial cell line BV-2, which was derived from primary mouse microglial cells. We investigated the effects of aprepitant on the viability of BV-2 microglia. Aprepitant showed little cytotoxic effect at concentration ranging from 1 to 8 μM (Figure 3A, *p* > 0.05). Subsequently, we investigated the effect of aprepitant on LPS-induced microglia activation and expressions of proinflammatory cytokines in BV-2 cells. BV-2 cells were co-cultured with LPS (1 μg/ml) and aprepitant (2 μM) for 16 h. Treatment with aprepitant significantly suppressed LPS-induced over-expression of IBA-1 (Figure 3B, *p* < 0.01), in BV-2 microglia. In contrast with treatment with LPS alone, aprepitant (2 μM) significantly suppressed LPS-induced MCP-1, IL-1β, TNF-α and IL-6 mRNA expression in BV-2 cells (Figure 3C, *p* < 0.01). In addition, aprepitant (2 μM) decreased the LPS-induced secretion of MCP-1, IL-1β, TNF-α and IL-6 in BV-2 (Figure 3D, *p* < 0.01). However, aprepitant (2 μM) alone showed no marked effects on the mRNA and protein expression of MCP-1, IL-1β, TNF-α and IL-6 in BV-2 cells (Figures 3C,D, *p* > 0.05). These results demonstrated that aprepitant could inhibit LPS-induced microglial activation.

Aprepitant inhibited LPS-induced p38 and JNK phosphorylation, but not ERK1/2 phosphorylation in BV2 microglia.

**FIGURE 4:**
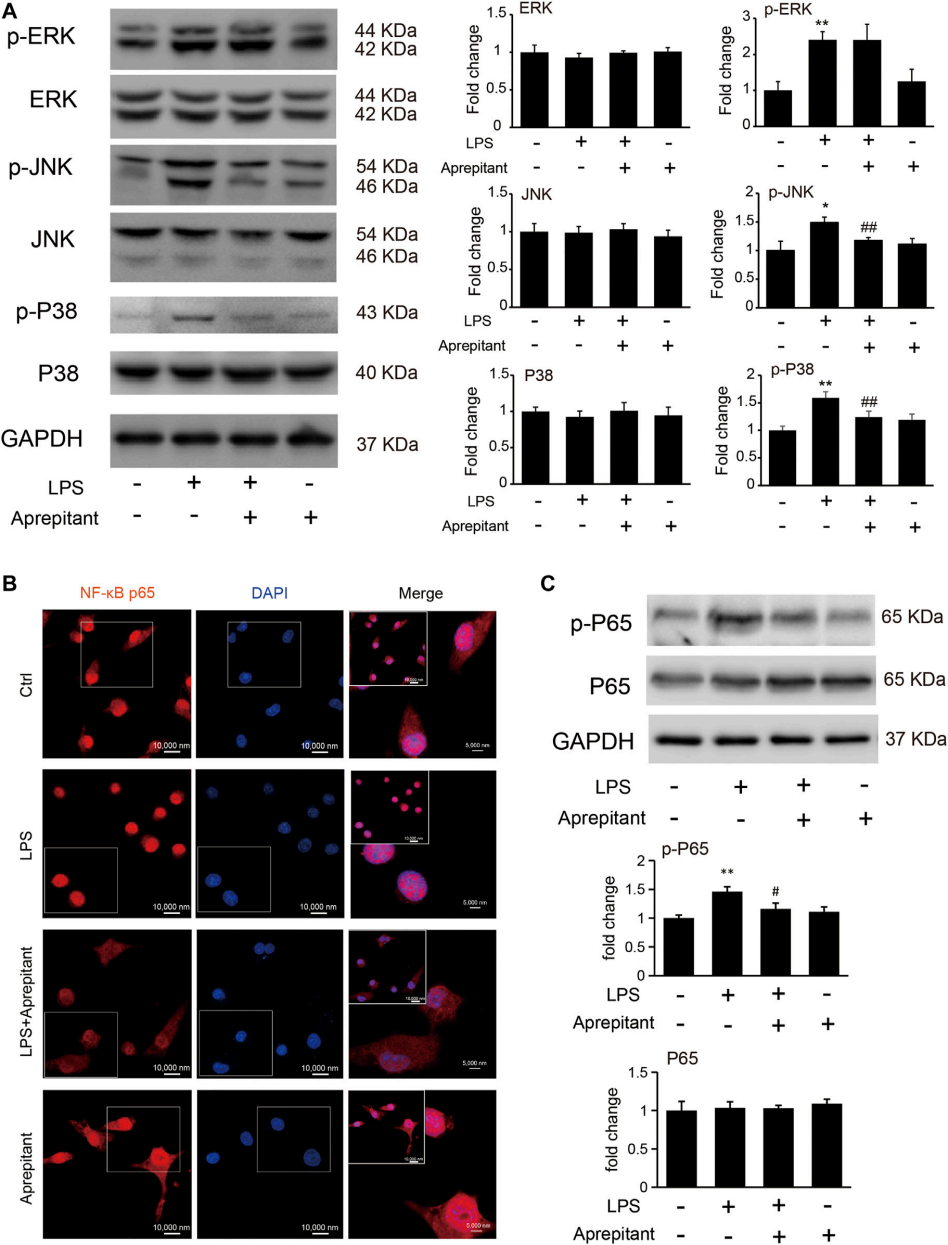
Effects of aprepitant on the phosphorylation of MAPK and nuclear translocation of NF-κBp65 in BV-2 microglia. **(A)** The protein and phosphorylation of ERK, JNK and p38 MAPK in BV-2 microglia. **(B)** Immunofluorescence was used to analyze NF-κBp65 nuclear translocation. **(C)** The phosphorylation and protein expression of NF-kBp65 in BV-2 microglia were assessed by western blotting (*n* = 6). Data are expressed as mean ± SEM. One-way ANOVA, *p < 0.05, **p < 0.01 versus control, #*p* < 0.05, ##*p* < 0.01 versus LPS.

To clarify the mechanisms underlying the anti-inflammatory effect of aprepitant, we assessed the effects of aprepitant on LPS-induced phosphorylation of MAPK in BV-2 cells. The results revealed that aprepitant (2 μM) significantly suppressed LPS-induced up-regulated of phosphorylation of p38 mitogen-activated protein kinase (p38) and c-Jun NH2 terminal protein kinase (JNK) (Figure 4A, *p* < 0.05), in BV-2 microglia. However, treatment with aprepitant (2 μM) had no influence on LPS-induced up-regulated of phosphorylation of extracellular signal-regulated kinase-1/2 (ERK1/2) in BV-2 (Figure 4A, *p* > 0.05). Furthermore, LPS treatment induced the translocation from the cytoplasm to the nucleus (Figure 4B) and increased of nuclear factor κB p65 (NF-κBp65) phosphorylation level (Figure 4C, *p* < 0.05). Compared with the LPS-treated group, co-incubation with aprepitant (2 μM) significantly reduced the effect (Figures 4B,C, *p* < 0.01).

### The JNK, and p38/MAPK were Involved in the Analgesic Mechanisms of Aprepitant

**FIGURE 5:**
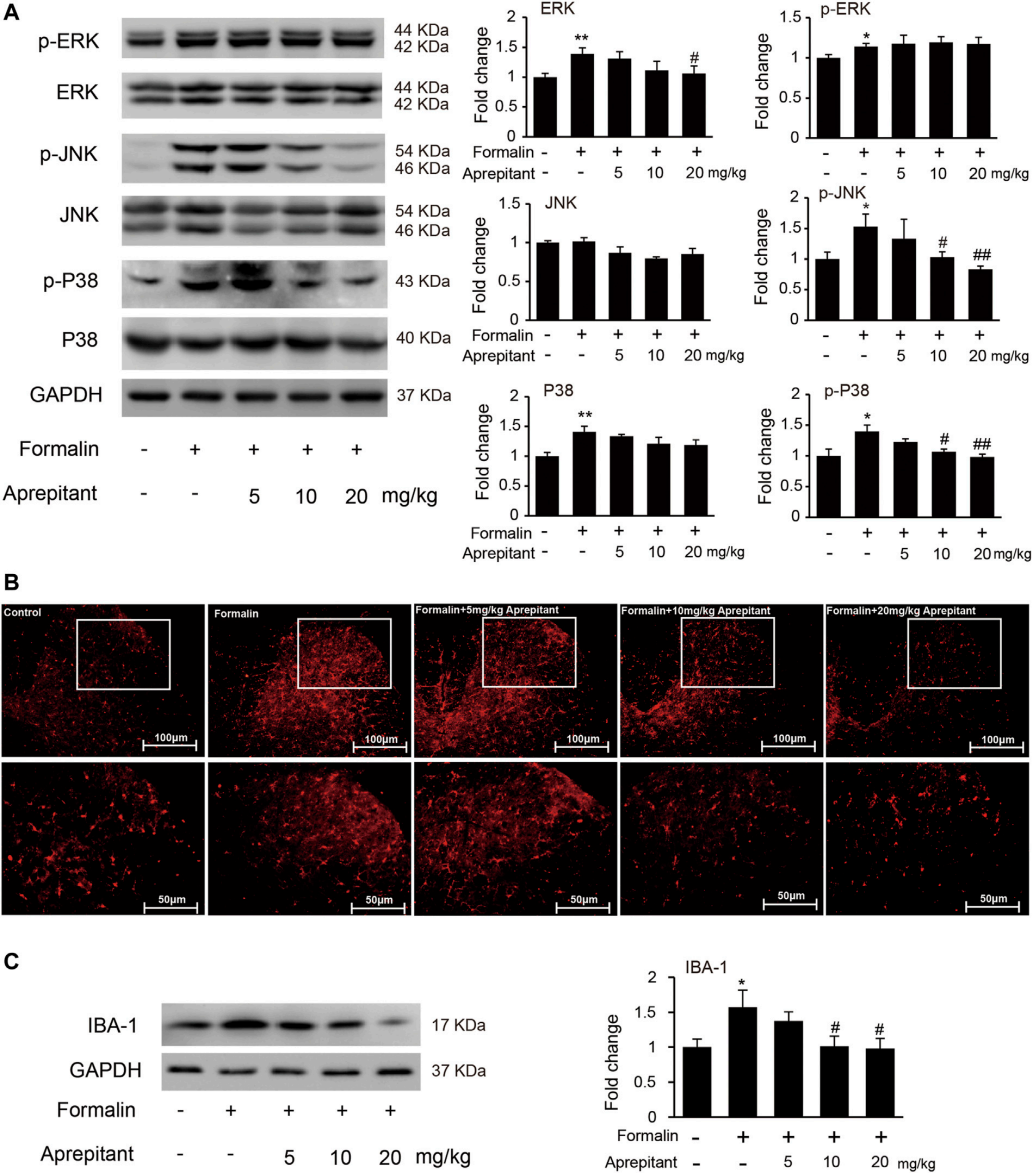
Effects of aprepitant on the phosphorylation of MAPK and microglial activation in spinal cord of mice with formalin-induced inflammatory pain. **(A)** The protein and phosphorylation of ERK, JNK and p38 MAPK in the spinal cord. **(B)** Confocal images of immunostaining showed the expression of IBA-1 in the spinal cord. **(C)** Western blot analysis of the expression of IBA1 in the spinal cord (*n* = 4). Data are expressed as mean ± SEM. One-way ANOVA, *p < 0.05, **p < 0.01 versus control, #*p* < 0.05, ##*p* < 0.01 versus formalin.

To determine whether the analgesia effect of aprepitant on mouse paw injected with formalin was associated with the inhibition of JNK and p38/MAPK, we evaluated phosphorylation of ERK1/2, JNK, and p38/MAPK in mouse spinal cord. Compared to the control group, the phosphorylation of ERK1/2, JNK, and p38/MAPK in the formalin group were significantly increased (Figure 5A, *p* < 0.05). Aprepitant effectively inhibited the formalin-induced phosphorylation of JNK, and p38/MAPK in the spinal cord at L4-L5 segments (Figure 5A, *p* < 0.05). Consistent with previous results, the mice with formalin injection had significant microglial cell activation, which was manifested as an increase in the expression of the microglial marker IBA-1 in the spinal cord at L4-L5 segments. However, co-treatment with aprepitant (10 mg/kg, 20 mg/kg) significantly reduced these pathological changes (compared with the formalin alone group, Figures 5B,C, *p* < 0.05).

**FIGURE 6:**
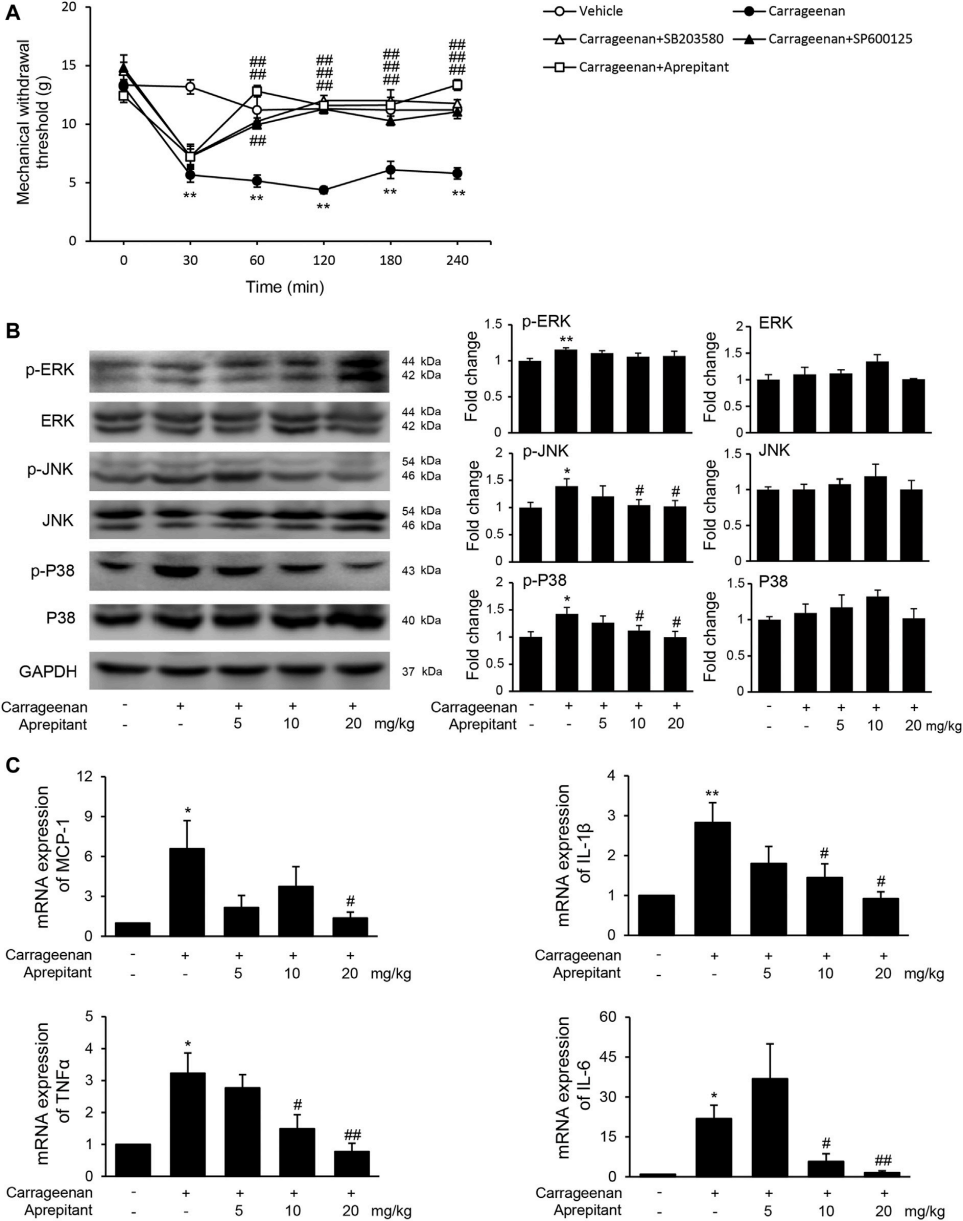
Effect of aprepitant on carrageenan-induced mechanical allodynia and microglial activation in the spinal cord. **(A)** Withdrawal threshold in treatment groups versus control mice. Eleven to twelve mice were included in each of the groups. Data are expressed as mean ± SEM. One-way ANOVA, **p < 0.01 versus control, ##*p* < 0.01 versus carrageenan. **(B)** The representative immunoblots and graphic representation of relative protein and phosphorylation of ERK, JNK and p38 MAPK in spinal cord. **(C)** The mRNA expression of MCP-1, IL-1β, TNF-α and IL-6 in spinal cord of mice with carrageenan-induced inflammatory pain (*n* = 4). Data are expressed as mean ± SEM. Two-way ANOVA, *p < 0.05, **p < 0.01 versus control, #*p* < 0.05, ##*p* < 0.01 versus carrageenan.

We also evaluated the effects of aprepitant on pain behavior, and phosphorylation of MAPK in mice with carrageenan-induced inflammatory pain (Figure 6). As shown in Figure 6A, the pain behavioral test demonstrated that treatment with aprepitant via the intrathecal (2 nmol, i.t.) route remarkably alleviated the pain behavior induced by carrageenan (Figure 6A, *p* < 0.05). The p38 inhibitor (SB203580, 10 nmol, i.t.) and JNK inhibitor (SP600125, 5 μg, i.t.) showed similar effects, reversing carrageenan-induced decreased of mechanical withdrawal threshold (Figure 6A, *p* < 0.01). Aprepitant markedly reduced phosphorylation of JNK and p38/MAPK in the spinal cord in mice with carrageenan-induced inflammatory pain (Figure 6B, *p* < 0.01). In addition, the administration of aprepitant suppressed the expression of pro-inflammatory cytokines induced by carrageenan in the spinal cord (Figure 6C, *p* < 0.05).

## Discussion

**FIGURE 7:**
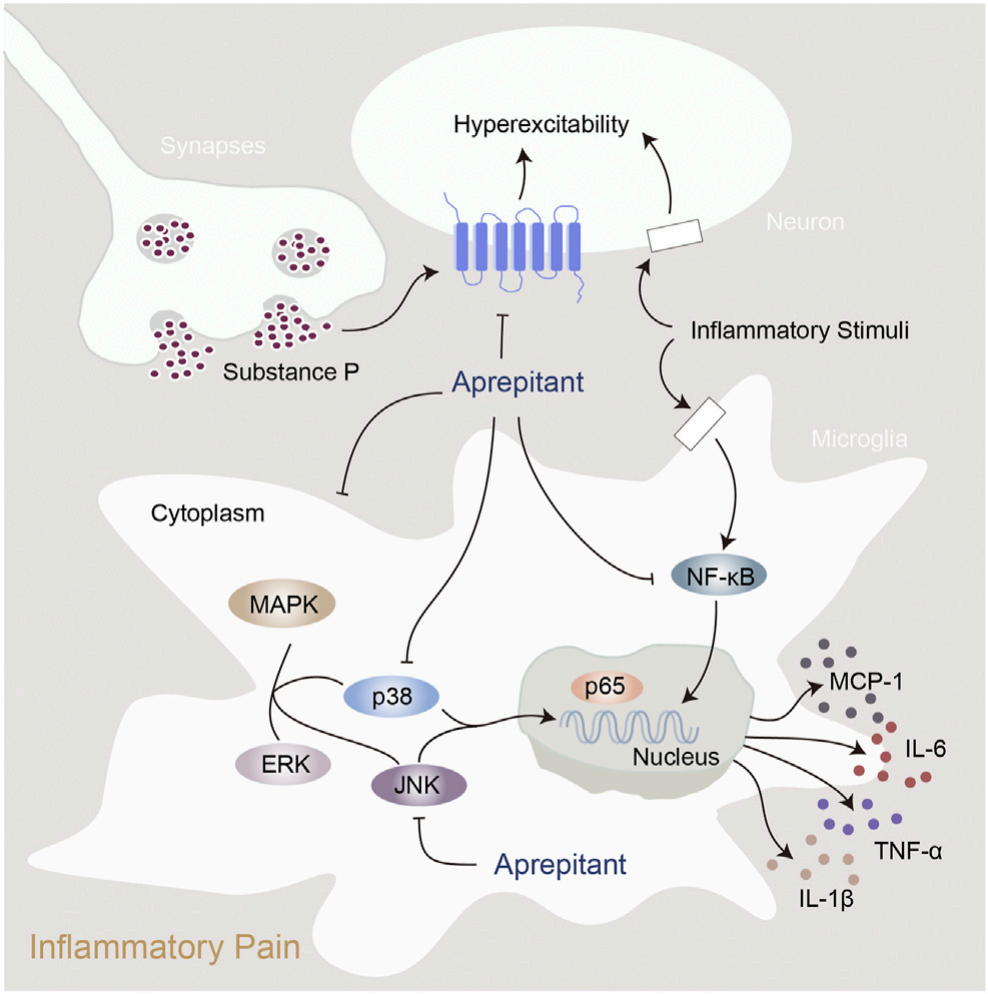
Proposed mechanisms of action by which aprepitant inhibited inflammation in mice with inflammatory pain.

In the present study, we found that aprepitant suppressed the excitability of dorsal root ganglion neurons and alleviated formalin-induced pain behavior. Furthermore, aprepitant application suppressed the LPS-induced activation in BV-2 microglial cells. In addition, aprepitant significantly down-regulated the phosphorylation of JNK and p38/MAPK, over-translocation into nucleus of NF-κBp65, as well as the increased of proinflammatory cytokines. Similarly, the administration of aprepitant inhibited pain behavior, microglial activation and the phosphorylation of JNK and p38/MAPK in mice with carrageenan-induced inflammatory pain. Therefore, aprepitant is a potential therapeutic approach to alleviate the inflammatory pain mediated by overactivation of microglia. Model of the mechanism underlying inflammatory pain and aprepitant-induced inhibition on inflammatory pain is illustrated in Figure 7.

Inflammatory pain refers to pain associated with tissue damage and inflammation of any cause, such as trauma, infection, and heat. The most common treatments for inflammatory pain include NSAIDs and opioids (Tomic et al., 2018; Gomes et al., 2020). Although these drugs are therapeutically effective at some time, they are associated with numerous side effects, such as liver damage, kidney damage and severe gastrointestinal reactions (Tomic et al., 2018). Aprepitant is widely used for the prevention of chemotherapy-induced nausea and vomiting in clinic. In the electrical hyperalgesia model in human, aprepitant has no therapeutical effects on measures of central sensitization (Chizh et al., 2007). However, a growing number of studies indicate that aprepitant has analgesic and anti-inflammatory effects (Ogawa et al., 2016; Liu BK. et al., 2019; Munoz and Covenas, 2020), there is little research on the influences of aprepitant in inflammatory pain. Thus, we investigated the effects of aprepitant on inflammatory pain, and explored the underlying mechanisms.

The data we presented from dissociated DRG neurons showed that aprepitant led to inhibition of the neuronal excitatory. Using the mice model with formalin-induced inflammatory pain, we found that aprepitant administration significantly decreased the time of licking/biting compared with the mice with formalin application alone. In addition, the threshold of mechanical allodynia, markedly reduced in formalin mice, was increased with aprepitant administration, indicating that aprepitant treatment relieved pain. Based on the potent anti-inflammatory effect of aprepitant on peripheral inflammatory conditions (Ramirez-Garcia et al., 2019), and its remarkable ability to inhibit mechanical allodynia, it is reasonable to believe that aprepitant may also alleviate inflammatory pain through reduction of central inflammation. In line with previous studies, our results suggested aprepitant remarkably decreased inflammatory cell infiltration, and increased the latency to paw withdrawal in mice with inflammatory pain.

Neuroinflammation mediated by microglia is involved in the pathogenesis of inflammatory pain (Inoue and Tsuda, 2018; Ji et al., 2018). Microglia is an important component that plays a critical role in the initiation, maintenance, and resolution of chronic pain in the central nervous system (Wolf et al., 2017; Chen et al., 2018; Inoue and Tsuda, 2018). Microglia activation is initiated in response to various extracellular stimuli such as LPS and inflammatory cytokines (George et al., 2019). Activated microglia cells release abundant inflammatory mediators, including IL-1β, IL-6, TNFα, and MCP-1, which can activate nociceptors directly or increase the excitability of neurons (Salter and Stevens, 2017; Inoue and Tsuda, 2018; Moehring et al., 2018). Administration of aprepitant could down-regulate the formalin-induced expressions of IL-1β, IL-6, TNFα, and MCP-1 in mice.

Exposure to LPS can lead to the activation of BV-2 microglial cells and induce inflammatory responses (Liao et al., 2020). In this study, aprepitant suppressed the LPS-induced expression of IBA1, and also inhibited LPS-induced expressions of inflammatory cytokines in BV-2 microglial cells. MAPK signaling pathway is activated to induce inflammation and the expression of proinflammatory mediators (Zhao et al., 2017; Ji et al., 2018). Microglial p38 is primarily and dramatically activated in the spinal dorsal horn (Gao and Ji, 2010). The p38 inhibitor significantly attenuates pain behaviors and proinflammatory cytokines synthesis (Jin et al., 2003; Gao and Ji, 2010). JNK plays an important role in inflammatory response in microglia (Subedi et al., 2019). It has been reported that JNK inhibitor can significantly alleviate persistent inflammatory pain and microglial activation (Subedi et al., 2019). The inhibition of NK1R expression can decrease the phosphorylation levels of MAPKs (Erk1/2, JNK, and p38) (Fang et al., 2012). We found that aprepitant treatment remarkably down-regulated the increased phosphorylation of JNK and p38 *in vitro* and *in vivo*. These data suggested that the JNK and p38 were involved in the analgesic effects of aprepitant on inflammatory pain.

The activation of JNK and p38 can initiate the NF-κBp65 signaling (An et al., 2019; Subedi et al., 2019; Wu et al., 2019). In mice with intracerebral hemorrhage, aprepitant can downregulate PKC/p38/NFκB signaling pathway (Jin et al., 2021). In addition, the inhibition of NF-κBp65 can reverse the production and expression of pro-inflammatory mediators following carrageenan or LPS treatment (Alam et al., 2019). In fibroblast-like synoviocytes, aprepitant can inhibit TNFα-induced phosphorylation and degradation of IκBα, the inhibitor of NF-κBp65, as well as attenuate TNFα-induced nuclear translocation and activity of NF-κBp65 (Liu X. et al., 2019). In colorectal cancer, aprepitant can inhibit the NF-κB via PI3K/AKT axis (Ghahremanloo et al., 2021). These data suggest that the activity of NF-κBp65 may play an important role in the analgesic effect of aprepitant on inflammatory pain. Our results revealed that aprepitant effectively reversed the LPS-induced over-translocation into nucleus of NF-κBp65. Hence, it appeared that the inhibition of NF-κBp65 activation by aprepitant may be a possible mechanism underlying its inhibitory effect in microglia, which is critical for the suppression of the production of proinflammatory cytokine. Aprepitant might inhibit the activation of JNK, p38/MAPK and NF-κBp65 to attenuate inflammation in microglia and suppressed inflammatory pain in mice.

Aprepitant is currently licensed NK-1 antagonist both in the United Kingdom and United States. Apart from its role in prevention of chemotherapy-induced nausea and vomiting, aprepitant has been implicated in the regulation of many physiological and pathophysiological processes, such as postoperative emesis, gastric motility disorders, depression, itch, cough and cancer. This study demonstrated a novel role for aprepitant in the therapy of inflammatory pain through mechanisms associated with the inhibition of multiple targets in activated microglia in the spinal cord. Our results supported the potential for repurposing this agent for the treatment of inflammatory pain. However, the therapeutic potential of aprepitant has not been fully explored in animals or humans. Future studies are needed to evaluate the efficacy of NK-1R antagonists in the pharmacotherapy practice for the treatment of diseases.

## Data Availability

The original contributions presented in the study are included in the article/Supplementary Material, further inquiries can be directed to the corresponding authors.
